# Parental Nutritional Knowledge and Type of Diet as the Key Factors Influencing the Safety of Vegetarian Diets for Children Aged 12–36 Months

**DOI:** 10.3390/nu15102244

**Published:** 2023-05-09

**Authors:** Malgorzata Kostecka, Julianna Kostecka, Izabella Jackowska, Katarzyna Iłowiecka

**Affiliations:** 1 Faculty of Food Science and Biotechnology, University of Life Sciences, Akademicka 15, 20-950 Lublin, Poland; izabella.jackowska@up.lublin.pl; 2 Faculty of Medicine, Medical University of Lublin, Chodźki 19, 20-093 Lublin, Poland; kostecka.julianna@gmail.com; 3 Department of Food and Nutrition, Medical University of Lublin, Chodźki 4a, 20-093 Lublin, Poland; katarzyna.ilowiecka@umlub.pl

**Keywords:** vegetarian, parental knowledge, diet

## Abstract

There are some concerns about the adequacy of vegetarian diets for pregnant women, infants, and young children because diets that exclude meat and other animal-based products increase the risk of nutrient deficiencies. The aim of the present study was to assess the nutritional knowledge of parents raising 12- to 36-month-old children on vegetarian diets and to evaluate the children’s diets based on the recommendations formulated in the model food ration. The study involved a questionnaire survey that was completed by 326 women raising their children on various types of vegetarian diets and 198 women raising their children on an omnivorous diet. Mothers raising children on a lacto-ovo-vegetarian diet had the highest nutritional knowledge scores (15.8 points on average), whereas control group mothers and women raising children on a vegan diet had the lowest nutritional knowledge scores (average of 13.6 points). Parents who raised their children on more restrictive vegetarian diets were more aware of the risk of nutritional deficiencies and administered dietary supplements more frequently. A vegetarian diet can be safe for young children, but parents should be educated about the risk of nutritional deficiencies and the principles of healthy nutrition regardless of the administered diet, and effective communication between parents, pediatricians, and dietitians should be the cornerstone of every nutritional strategy in the management of vegetarian children.

## 1. Introduction

Young children’s diets not only satisfy their nutritional needs and promote healthy growth and development, but they also influence children’s long-term health and well-being, as well as the risk of diet-dependent diseases in adulthood [1]. There are some concerns about the adequacy of vegetarian diets for pregnant women, infants, and young children because diets that exclude meat and other animal-based products increase the risk of nutrient deficiencies [2,3,4]. The number of families following plant-based diets, including parents who raise their children on a vegetarian diet, has increased in recent years in developed countries [5]. In the European population, the estimated prevalence of vegetarian diets ranges from 1.2% to 1.5% in Portugal and Spain to 7% in the United Kingdom and 10% in Germany. New research has revealed that 1 in 12 parents are raising their children as vegan [6]. For adults, adherence to a diet that is free of meat or meat and other animal-based products is a conscious lifestyle choice [7], but young children are completely dependent on their parents, and it is the parents’ responsibility to ensure that their children’s diets are balanced and nutritious [8].

In its position statement of 2009, the Canadian Pediatric Society acknowledged that a well-balanced and appropriately supplemented vegetarian diet is supported by evidence and can be recommended for children, including toddlers and preschoolers [9,10]. The Canadian Pediatric Society also emphasized the importance of monitoring children’s protein and energy intake, and supplementing their diets when necessary [10]. According to North American guidelines, well-planned vegetarian and vegan diets are safe for people of all ages [10]. In 2016, the American Academy of Nutrition and Dietetics published its revised position paper on vegetarian diets [11], reiterating its conclusion statement from 2009 that these diets are appropriate for all stages of the life cycle, including pregnancy, lactation, infancy, childhood, and adolescence, with an additional note endorsing the environmental benefits of plant-based diets. The 2020–2025 Dietary Guidelines for Americans include a healthy vegetarian eating pattern for children. Eggs, dairy products, soy-based products, nuts, seeds, fruits, vegetables, and oils should be regularly administered to children aged 12–24 months if their diets do not include meat, poultry, and seafood. These guidelines also call for clinician involvement to monitor the adequacy of a vegetarian diet in childhood [12]. According to the Healthy Eating Guidelines For Your Vegetarian Toddler: 1–3 years, a specially planned vegetarian diet can be healthy for all ages, including toddlers [13].

The position paper on complementary feeding of the European Society for Pediatric Gastroenterology, Hepatology, and Nutrition [14] includes a dedicated section on vegan and vegetarian diets which emphasizes that complementary feeding of infants and young children should receive special attention, including regular medical and dietetic supervision. According to the German Nutrition Society (DGE), a balanced and varied ovo-lacto-vegetarian diet can meet the energy and nutrient requirements of young children and adolescents. Complete elimination of animal products is not recommended by the DGE as a permanent dietary pattern for adolescents [15]. The consequences of vitamin B12 deficiency resulting from the lack of supplementation, including neurological damage and potential death, were firmly placed within the remit of parental responsibility. The Spanish Pediatric Association [16] expressed a preference for omnivorous and lacto-ovo-vegetarian diets over vegan diets in infants and young children, and advocated obligatory vitamin B12 supplementation and continuous monitoring of risk nutrients in vegan children. The French-speaking Pediatric Hepatology, Gastroenterology, and Nutrition Group [17] adopted an even stronger stance and summarized its position by stating that a vegan diet is ‘not recommended for infants, children, and adolescents due to the risk of multiple nutritional deficiencies that are inevitable in the absence of supplements’ [17]. An equally restrictive approach has been adopted by the Nutrition Committee of the European Society for Pediatric Gastroenterology, Hepatology, and Nutrition (ESPGHAN), which stated in its position paper that vegan diets should be discouraged during complementary feeding of children younger than 12 months [18]. The position statement of the Polish Society of Pediatric Gastroenterology, Hepatology and Nutrition, revised in 2021, indicates that infants and young children raised on vegetarian diets require supplementation and regular consultations with nutrition specialists. Children can be placed on a vegan diet if their development and dietary intake are monitored, their diets are adequately supplemented, and the parents are aware that an unbalanced diet and lack of supplementation can have serious consequences for the child’s health or even life [19].

The model food ration proposed by Weker et al. [20] is a helpful tool in planning young children’s diets, both at the individual and group level. The model food ration is based on Polish nutritional guidelines, and it relies on six food groups. The model can be used to plan balanced diets that meet the child’s energy needs and nutritional requirements. Only diets characterized by an appropriate quantity and quality of food products from different groups can provide the nutrients required for healthy growth and development [21].

The aim of the present study was to assess the nutritional knowledge of parents raising 12- to 36-month-old children on vegetarian diets and to evaluate the children’s diets based on the recommendations formulated in the model food ration. The nutritional knowledge and eating habits of families raising toddlers on vegetarian and omnivorous diets were compared to evaluate nutritional deficiencies.

## 2. Materials and Methods

### 2.1. Study Design and Participants

The study involved a questionnaire survey that was completed by 326 women raising their children on various types of vegetarian diets and 198 women raising their children on an omnivorous diet (control group). Only mothers of children aged 12–36 months who had been fully transitioned to family foods and were raised on a vegetarian diet or an omnivorous diet were invited to participate in the study. The exclusion criteria were adherence to an elimination diet, metabolic diseases, allergies, and food intolerance in children.

The questionnaire was posted on social media channels in general discussion groups for parents and families adhering to a vegetarian diet, and it was also distributed among the patients of pediatric clinics in Lublin, Zamość, Rzeszów, and other cities in southeastern Poland between September 2021 and December 2022. The questionnaire was based on food frequency and nutritional knowledge questionnaires that had been previously designed by the authors [22,23]. During a pilot test, the questionnaire was completed by 50 respondents in July 2021, and the results were analyzed. The pilot test revealed that the questions were comprehensible and did not require modification. The questionnaire was fully anonymous, and the respondents were required to answer all questions. Paper questionnaires were filled out independently by the patients of pediatric clinics (104 women from the experimental group and 43 women from the control group), and the average completion time was 35 ± 11 min. The remaining respondents completed the online questionnaire. All questionnaires were fully completed, and they were qualified for further evaluation.

The questionnaire consisted of three parts. The first part contained six demographic questions; the second part contained 26 question testing the respondents’ knowledge of Polish nutritional guidelines for toddlers, and the third part contained 35 questions concerning children’s daily eating habits. Demographic questions were designed to collect information about the respondents’ age, place of residence, education, occupational status, and type of vegetarian or omnivorous diet (all respondents were female). The second part contained closed-ended, single-choice questions (the questions are presented in Table 3. In the third part, the respondents answered questions about their children’s diets; they responded regarding the consumption frequency of products belonging to the following food groups: cereal products, milk, dairy products, plant-based beverages, meat, poultry, fish, fruits, legumes, fats, sugar, sweets, baked products, sweet and savory snacks, fast food, water, sweetened beverages, carbonated soft drinks, hot beverages ([] never, [] 1–3 times per month, [] once a week, [] several times per week, [] once a day, [] several times a day), dietary supplements, consultations with a dietitian, and sources of nutritional knowledge. The consumption frequency of products belonging to six food groups (cereal products, protein foods, dairy products, vegetables, fruits, and fats) was evaluated on the following scale: [] below the recommended number of servings, [] recommended number of servings, and [] higher than the recommended number of servings relative to the model food ration.

### 2.2. Nutritional Knowledge

The second part of the questionnaire contained 26 nutritional knowledge questions, and the respondents could choose one of the following answers: true, false, do not know. One point was awarded for every correct answer, and zero points were awarded for every incorrect and “do not know” answer. The points were summed up for each respondent (range 0 to 26 points). Based on the distribution of points, the respondents were divided into three nutritional knowledge categories: low (0–13 points), average (14–20 points), and high (21–26 points). The questions were designed to test the respondents’ knowledge about nutritional guidelines for toddlers, meal planning guidelines, the number of servings in the model food ration, and the recommendations of the Polish Society of Pediatric Gastroenterology, Hepatology, and Nutrition (2021) concerning vegetarian diets, nutritional deficiencies, and consultations with a dietitian. The research plan was approved by the Ethics Committee of the Medical University of Lublin (KE-0254/273/2021).

### 2.3. Statistical Analysis

Categorical variables were presented as sample percentages (%), and continuous variables were expressed by median values and the interquartile range (IQR). The differences between groups were analyzed with the chi-squared test (categorical variables). The Wilcoxon signed-rank test for two dependent samples was used to comprehensively compare test data and retest data and to verify differences in mean food consumption frequency between the test and the retest. The odds ratios (ORs) and 95% confidence intervals (95% CIs) were calculated. The reference categories (OR = 1.00) were the recommended intake of product portions, the type of the diet, membership in the control group, and maternal nutritional knowledge scores (21–26 points). The ORs were adjusted for the diet and the frequency of serving specific food ingredients. The significance of ORs was assessed by Wald’s statistics. The results of all tests were regarded as statistically significant at *p* < 0.05. Data were processed in the Statistica program (version 13.1 PL; StatSoft Inc., Tulsa, OK, USA; StatSoft, Krakow, Poland).

## 3. Results

The questionnaire was completed by a total of 326 women raising children on a vegetarian diet, including 146 women raising children on a lacto-ovo-vegetarian diet (44.8%), 48 women raising children on a vegan diet (14.7%), 64 women raising children on an ovo-vegetarian diet (19.6%), 68 raising children on a lacto-vegetarian diet (20.9%), and 198 women raising children on an omnivorous diet (control group)—Table 1.

### 3.1. Nutritional Knowledge

The respondents’ nutritional knowledge scores are presented in Table 2 and Table 3. Mothers raising children on a lacto-ovo-vegetarian diet had the highest nutritional knowledge scores (15.8 points on average), whereas control group mothers and women raising children on a vegan diet had the lowest nutritional knowledge scores (average of 13.6 points). In the surveyed population, 45% of women were characterized by low levels of nutritional knowledge. Across the examined occupational groups, nutritional knowledge scores were lowest among unemployed mothers and women with a primary school education. University education and vocational education were factors that significantly differentiated nutritional knowledge scores in the general population (*p* = 0.027), the control group (*p* = 0.0.021), and the experimental group (*p* = 0.032).

An analysis of parental knowledge about the risk of nutritional deficiencies in children raised on a vegetarian diet produced completely different results. Considerable differences were observed between groups (*p* = 0.016), and mothers resorting to the most restrictive diets had the highest nutritional knowledge scores (72.9% of mothers raising their children on a vegan diet vs 57.6% of mothers raising their children on a lacto-ovo-vegetarian diet; *p* = 0.003).

**Figure 1:**
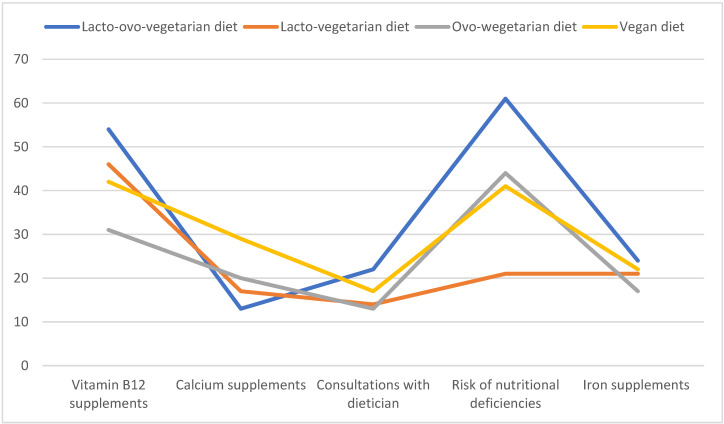
Number of mothers adhering to different types of vegetarian diets who administered dietary supplements and consulted dietitians.

Vegan mothers also more frequently administered calcium supplements [OR 1.36; 95% CI 1.07–1.56, *p* = 0.01] than women raising their children on a lacto-ovo-vegetarian diet. Vitamin B12 supplements were most frequently administered to children raised on a vegan diet, whereas mothers adhering to an ovo-vegetarian diet were least likely to administer vitamin B12 supplements [OR 0.78; 95% CI 0.64–0.91, *p*= 0.0078] or were least likely to administer other supplements. Iron supplements were least frequently administered by women adhering to a lacto-ovo-vegetarian diet; iron supplements were administered by only 16.4% of lacto-ovo-vegetarian mothers vs. 45.8% of vegan mothers (*p* = 0.014)—Figure 1.

### 3.2. Dietary Habits in Reference to the Model Food Ration

The respondents’ dietary habits were also evaluated in the questionnaire (Table 4). In the vegetarian group, the consumption of fruits (OR 1.34; 95% CI 1.11–1.48, *p* < 0.05) and vegetables (OR 1.46; 95% CI 1.25–1.72, *p* < 0.01) was significantly higher than in the control group. The type of vegetarian diet influenced the consumption frequency of fruits, legumes, cereals, sweets, and sweet snacks, and these foods were least frequently consumed by children on a vegan diet (OR 1.36; 95% CI 1.03–1.49; *p* < 0.01). The frequency of juice consumption was also lowest among vegan children (once a day in the lacto-ovo-vegetarian group vs several times per week in the vegan group; *p* < 0.01). Sweets were consumed with similar frequency in all groups. Natural dairy products and plain yogurt were consumed significantly more often by control group children (once a day vs several times per week in the vegetarian group; *p* < 0.05), children raised on a lacto-ovo-vegetarian diet (OR 1.39; 95% CI 1.01–1.54, *p* < 0.05), and children raised on a lacto-vegetarian diet (OR 1.36; 95% CI 1.05–1.46, *p* < 0.05) than vegan children.

The type of diet did not induce differences in water intake. Only the children of mothers with low nutritional knowledge scores drank water significantly less often (several times a week) than the children of mothers with high nutritional knowledge scores (several times a day; *p* < 0.01). Maternal nutritional knowledge significantly influenced food choices, consumption frequency of recommended foods (significantly lower consumption of vegetables and dairy products), and sweet snacks. Sweet snacks were most frequently consumed (once a day/several times a day) by the children of mothers with low nutritional knowledge scores (OR 1.45; 95% CI 1.09–1.62, *p* < 0.01).

An analysis of the consumption frequency of different product groups relative to the recommendations formulated in the model food ration demonstrated that both the type of diet and maternal nutritional knowledge influenced the composition of diets served to children aged 12–36 months (Table 5).

## 4. Discussion

A vegetarian diet is an alternative diet. A vegetarian diet excludes certain product groups that could pose a threat to the health of growing children, thus raising concerns about the safety and rationale behind vegetarian diets. Plant-based diets may be deficient in energy and nutrients, and vegetarian substitutes for animal-based products do not always effectively address these deficiencies.

### 4.1. Model Food Ration and Vegetarian Diets

Plant-based diets are becoming increasingly popular in Western societies, and they often lead to milk restriction. In very young children, the replacement of milk with other foods without proper nutritional advice may lead to nutritional deficiencies and, consequently, impaired growth and development [24]. Plant-based milk substitutes can deliver health benefits due to their antioxidant properties and a favorable fatty acid profile, which decreases the risk of cardiovascular disease, cancer, and diabetes [25]. However, these products could also be detrimental to health because they are low in wholesome proteins and contain antinutritional factors, such as phytates and oxalates, which decrease the bioavailability of minerals (zinc and calcium) and vitamins [26]. Plant-based beverages are usually sweetened to enhance taste acceptance, which exerts a negative impact on oral health, in particular in infants and young children. According to the WHO (2016), the consumption of added sugar plays an important role in the etiology of dental caries in children [27]. A 2019 report published by the Robert Wood Johnson Foundation-Healthy Eating Research (RWJF-HER) summarized the benefits and risks of plant-based milk for young children. According to a consensus statement developed in a collaborative effort by the American Academy of Pediatrics, Academy of Nutrition and Dietetics, American Heart Association, and American Academy of Pediatric Dentistry, there is little evidence to indicate that plant-based beverages deliver greater health benefits for young children than cow’s milk, and frequent consumption of plant-based beverages can pose a potential health risk if the overall nutrient content of the child’s diet is not carefully managed [28,29]. There is considerable evidence to indicate that the replacement of cow’s milk or infant formulas with plant-based beverages (past the age of 12 months) can lead to malnutrition and kwashiorkor in young children, especially if these beverages are the only or the main source of protein [30,31,32]. In the studied population, half of the mothers replaced cow’s milk with plant-based beverages in their children’s diets.

According to the model food ration, fats, including fats rich in PUFAs, especially EPA and DHA, should be incorporated into children’s diets. In the analyzed population, the consumption of added fat, including vegetable oil, olive oil, butter, and margarine, was associated with the type of diet. Fat consumption was highest in the lacto-ovo-vegetarian group and lowest in the vegan group, which corroborates the findings of other authors [33,34]. The above applies particularly to saturated fats and long-chain omega-3 fatty acids which are found mainly in fish, seafood, and eggs [35]. Vegan diets are particularly abundant in omega-6 fatty acids, which can inhibit the conversion of the linolenic acid precursor to DHA and EPA [36].

The maximum intake of dietary fiber recommended by the American Academy of Pediatrics is 0.5 g/kg/day [37]. According to Polish guidelines, children should consume 9–19 g of dietary fiber/day in five daily servings of cereal products and vegetables. However, the fiber intake of some vegetarian children may exceed the recommended level three times [38]. Excessive fiber intake could pose a health risk for young children because high-fiber diets have low energy density, which may lead to inadequate calorie intake and decrease mineral absorption [37]. In the studied population, the intake of cereal products as an important source of dietary fiber was significantly lower in vegetarian than omnivorous children, but fiber intake in the daily food ration was not quantified.

### 4.2. Nutritional Deficiencies in Vegetarian and Vegan Children

Vegetarian diets can be deficient in the quantity and quality of some nutrients, including protein; iron; zinc; selenium; calcium; riboflavin; vitamins A, D, and B12; and essential fatty acids. Despite the fact that many omnivorous children are characterized by a low nutritional status and low intake of some nutrients (such as vitamin D and iron), vegan children are particularly at risk of nutritional deficiencies due to insufficient dietary nutrient intake and/or excessive intake of fiber and other plant-based dietary components that decrease the bioavailability of nutrients.

Elliott et al. [39] studied 248 vegetarians with a mean age of 2.2 years (SD 1.5) and found that adherence to a vegetarian diet was not linked with BMI, height-for-age z-score, or serum ferritin and 25-hydroxyvitamin D levels. However, vegetarian children were at greater risk of being underweight (zBMI < −2). Thane and Bates [40] included 1351 children (age: 1.5–4.5 years) from the National Diet and Nutrition Survey in their study to analyze and compare the dietary intakes and nutrient status of vegetarian and non-vegetarian Asian preschoolers. The cited authors concluded that lower fat and sodium intake and a higher intake of antioxidant vitamins may be beneficial, but they observed that low serum ferritin levels may be a potential risk for growth impairment.

A study of vegan preschoolers and school children revealed adequate iron intake [41]. However, due to differences in iron bioavailability, the demand for iron is 1.8 times higher in vegans and lacto-ovo-vegetarians than in non-vegetarians [42]. In the present study, iron supplements were most frequently administered to children by mothers adhering to a vegan diet. Vitamin C and other nutrients found in vegetables enhance the absorption of non-heme iron [43,44], which in turn can be inhibited by some plant compounds. Parents should be familiar with iron-rich foods and should incorporate these products, in particular iron-fortified cereal products/breakfast cereals, dried beans, and peas, in their children’s diets because iron deficiency is the most common nutritional disorder in children [45,46]. Iron supplementation may be required in periods of rapid growth, especially in vegan children, but supplements should be consulted with a physician [46]. Vegetarian diets are abundant in phytates, which decrease the absorption of iron and zinc. However, iron and zinc deficiencies are not very common in developed countries. Vegan children should be monitored for vitamin B12, iron, and zinc status [47].

According to Rudloff [48] and Ambroszkiewicz et al. [49], calcium intake is approximately 50% lower in children consuming a vegetarian diet than in omnivorous children. Lacto- and lacto-ovo-vegetarians have a high intake of dairy products, and they are unlikely to be deficient in calcium [10]. In the studied group, the intake of milk and dairy products was consistent with recommendations only in lacto-ovo-vegetarian children who consumed ≥3 servings of dairy products per day. In turn, only 8.3% of vegan parents were familiar with the recommended daily intake of dairy products for children. According to research, adequate intake of calcium-rich foods, including soybeans, cereals, juice, and leafy vegetables with low oxalate content (Chinese cabbage, kale, Savoy cabbage) are good sources of highly available calcium for older children [10,38,50].

Vegetarians, in particular vegans, are at risk of vitamin B12 deficiency. In the examined population, more than 75% of the parents were aware of that risk, and vitamin B12 supplements were administered by lacto-ovo-vegetarian parents as well as 87.5% of vegan parents. In a study by Jeitler [51], all vegan parents administered vitamin B12 supplements to their children, as opposed to only 50% of vegan parents surveyed in 2003 [52]. According to nutritional guidelines, dairy products, soybeans, and eggs are good sources of vitamin B12 in a lacto-ovo-vegetarian diet, provided that these foods are consumed on a regular basis [53,54,55,56]. However, the intake of fortified foods and/or dietary supplements should be regularly monitored in vegan infants, children, and adolescents. Vegan children should consume at least three servings of food products rich in vitamin B12 per day or they should receive vitamin B12 supplements in the daily amount of 5–25 µg [48,57]. A manifested vitamin B12 deficiency should be initially treated with a single intramuscular injection of 1000 μg [48].

### 4.3. Nutritional Knowledge and the Safety of Vegetarian Diets

Many young vegetarian parents administer their diets to children [58]. These parents could place their children on vegetarian diets without being adequately educated and informed about the potentially negative health effects of inadequately planned diets in children.

The nutritional knowledge of parents raising children on a vegetarian diet remains insufficiently researched, but studies investigating the nutritional knowledge of adults have shown that the level is higher in vegetarians than in omnivores [51,59], which is consistent with the present findings. However, there is a general scarcity of studies investigating the relationship between the nutritional knowledge of vegetarian parents and the nutritional status of infants and toddlers. In the work of Jeitler [51], vegan participants with an average nutritional knowledge score of 3.3/5 points were better informed than vegetarians (2.5/5) and omnivores (2.2/5). According to the German Federal Institute for Risk Assessment, vegans have a high interest in nutrition and, therefore, high levels of nutritional knowledge [43]. However, despite high levels of general nutritional knowledge, not all vegans were familiar with the recommendations of nutrition societies, and similar observations were made in the present study. Jeitler [51] demonstrated that vegan participants were less familiar with the recommendations of the German Nutrition Society and the US Academy of Nutrition and Dietetics than could be expected based on their general nutritional knowledge. The present study also demonstrated that parental knowledge about nutritional deficiencies and supplementation in vegetarian children was significantly influenced by the type of diet [51]. Therefore, plant-based diets should be consulted with a physician and a dietitian. In the studied population, only 34.0% of the mothers informed pediatricians that their children adhered to a vegetarian diet, and only 20.2% of the parents consulted a dietitian at least once. Most of the parents surveyed by Bivi [8] also failed to inform pediatricians of their decision to exclude animal-based products from their children’s diets. Similar results were reported by Baldassarre et al. in a recent study of 360 Italian families, where 22.7% of the parents placed their children on alternative diets (semi-vegetarian, lacto-ovo-vegetarian, vegan) without consulting their pediatrician [4]. Parents who work closely with pediatricians and dietitians can effectively prevent nutritional deficiencies and minimize the risk of physical and mental dysfunctions in their children. Communication between parents and physicians should be the cornerstone of nutritional strategies in the management of vegetarian toddlers and preschoolers. Efforts should be made to enhance the nutritional knowledge of pediatricians because an unsupervised vegetarian or vegan diet can cause severe nutritional deficiencies with possible detrimental long-term effects [4].

### 4.4. Strengths and Limitations

The strength of the study was the large number of mothers raising toddlers on different types of vegetarian diets, in particular a vegan diet, which is highly restrictive and difficult to balance. To the best of the authors’ knowledge, this is the first Polish study where the relationships between parental nutritional knowledge and the eating habits of children raised on plant-based diets were examined on such a large and diverse population. Studies undertaken to identify the potential mistakes made by vegetarian parents provide valuable insights for nutrition education programs because nutritional deficiencies in children should be eliminated and rectified as early as possible. Another strength of the study was that it evaluated the consumption frequency of different food groups recommended by the model food ration, which applies to both omnivorous and vegetarian diets.

The main limitation of the present study and our previous research was that the questionnaires were not completed by fathers. As a result, paternal knowledge about nutrition and its influence on the safety of the children’s diets could not be evaluated. The limitations of the study included a qualitative approach to the daily food ration and a lack of food diaries for assessing nutritional deficiencies in children raised on different types of vegetarian diets. These issues will be addressed in subsequent stages of the research project, which commenced in January 2023 and is scheduled for completion in March 2024.

## 5. Conclusions

Lacto-ovo-vegetarian mothers were characterized by the highest nutritional knowledge scores and were most familiar with the nutrition guidelines for toddlers.

Parents who raised their children on more restrictive vegetarian diets were more aware of the risk of nutritional deficiencies and administered dietary supplements more frequently.

Maternal nutritional knowledge and the type of vegetarian diet significantly influenced the frequency with which children consumed various food products, excluding sweets.

Vegan mothers were most likely to depart from the nutritional recommendations formulated in the model food ration for children aged 12–36 months.

A vegetarian diet can be safe for young children, but parents should be educated about the risk of nutritional deficiencies and the principles of healthy nutrition regardless of the administered diet.

Effective communication between parents, pediatricians, and dietitians should be the cornerstone of every nutritional strategy in the management of vegetarian children.

## Figures and Tables

**Table 1:** Description of the studied sample.

Indicator		Total, *n* [%]	*p*-Value	Vegetarian Diet, *n* [%]	Omnivorous Diet (Control Group), *n* [%]
Gender	Female	524 [100%]	ns	326 [62.2%]	198 [37.8%]
Age [years]	18–25	71 [13.5%]	0.0038	37 [11.3%]	34 [17.2%]
	26–30	167 [31.8%]		111 [34.0%]	56 [28.3%]
	31–35	184 [35.1%]		113 [34.8%]	71 [35.8%]
	>35	102 [19.6%]		65 [19.9%]	37 [18.7%]
Place of residence	Rural area	46 [8.8%]	0.0014	28 [8.5%]	18 [9.1%]
	City with a population of up to 50,000	124 [23.7%]		79 [24.1%]	45 [22.7%]
	City with a population greater than 50,000	354 [67.5%]		219 [67.4%]	135 [68.2%]
Education	Primary school	16 [3.1%]	0.0025	9 [2.8%]	7 [3.5%]
	Vocational school	27 [5.1%]		15 [4.6%]	12 [6.0%]
	Secondary school	196 [37.4%]		131 [40.1%]	65 [32.8%]
	University	285 [54.4%]		171 [52.5%]	114 [57.7%]
Occupational status	Employed	338 [64.5%]	0.0013	212 [65.0%]	126 [63.6%]
	Unemployed	149 [28.4%]		86 [26.4%]	63 [31.9%]
	Student	37 [7.1%]		28 [8.6%]	9 [4.5%]

The results are statistically significant at *p* < 0.05 (chi-squared test).

**Table 2:** Nutritional knowledge scores (in points) and factors contributing to differences in maternal nutritional knowledge in the overall population, in the control group, and in the experimental group, depending on the type of vegetarian diet.

					Type of Vegetarian Diet				
	Total Sample	Control Group	Experimental Group	*p*-Value	Lacto-	Ovo-	Lacto-Ovo-	Vegan	*p*-Value
Median score	14.1 (7.4–21.3)	13.6 (8.2–19.6)	14.9 (7.4–21.3)	0.027	15.1 (8.6–19.2)	14.1 (7.4–17.8)	15.8 (8.1–20.7)	13.6 (8.5–21.3)	0.008
Nutritional knowledge score, *n* (%)									
Low	136	59	77		17	21	17	22	
Average	231	73	158		28	25	91	14	
High	157	66	91		23	18	38	12	
Occupational status									
Employed	14.6	14.1	14.2	ns	15.2	14.5	15.9	13.8	0.02
Unemployed	13.1	12.7	13.0	ns	14.1	11.9	13.8	13.1	ns
Student	15.2	14.6	16.5	0.002	15.9	14.3	16.5	13.5	0.003
Mother’s education									
Primary school	10.2	10.4	10.1	ns	9.5	-	10.3	-	ns
Secondary school	12.9	12.7	13.1	ns	8.7	12.3	12.5	12.3	ns
Vocational school	11.3	11.7	10.8	0.023	10.3	10.5	9	-	ns
University	16.8	14.9	15.7	0.001	15.9	14.6	17.1	13.4	0.0001

Low knowledge score (0–13 points), average knowledge score (14–20 points), high knowledge score (21–26 points), ns—not significant; *p*-value—significance of the observed differences between the control group and the experimental group and between lacto-ovo- and vegan groups (Mann-Whitney test) or test-retest differences (Wilcoxon test).

**Table 3:** Distribution of correct answers in the experimental group and the control group.

	All Vegetarian Groups	Control Group	*p*-Value	Lacto-Ovo-Vegetarian Group	Lacto-Vegetarian Group	Ovo-Vegetarian Group	Vegan Group	*p*-Value
Exclusive breastfeeding time recommended by the WHO	256 (78.5%)	124 (62.6%)	0.02	127 (86.9%)	54 (79.4%)	48 (75%)	27 (56.3%)	0.023
Age at which complementary foods should be introduced	211 (64.7%)	117 (59.1%)	0.031	95 (65.1%)	51 (75%)	43 (67.2%)	22 (45.8%)	0.037
Reasons for delaying the introduction of potentially allergenic foods	194 (59.5%)	107 (54.0%)	ns	112 (76.7%)	24 (35.3%)	39 (60.9%)	19 (39.6%)	0.021
The parent or caretaker decides what types of foods are served and when, but the child is free to decide whether and how much he/she wants to eat	197 (60.4%)	79 (39.9%)	0.018	72 (49.3%)	49 (72%)	47 (73.4%)	29 (60.4%)	0.035
Number of daily meals served to children aged 12–36 months	217 (66.5%)	124 (62.6%)	ns	118 (80.8%)	40 (58.8%)	27 (42.2%)	32 (66.7%)	0.038
Daily caloric intake of children aged 12–36 months	151 (46.3%)	94 (47.4%)	ns	71 (48.6%)	38 (55.9%)	20 (31.3%)	22 (45.8%)	0.041
Number of daily servings of dairy products	103 (31.6%)	134 (67.6%)	0.012	115 (78.7%)	23 (33.8%)	11 (17.2%)	4 (8.3%)	0.001
Number of daily servings of protein foods	112 (34.3%)	108 (54.5%)	0.015	48 (32.8%)	25 (36.7%)	27 (42.2%)	12 (25%)	0.023
Number of daily servings of cereal products	191 (58.6%)	114 (57.6%)	ns	72 (49.3%)	41 (60.3%)	46 (71.8%)	32 (66.7%)	0.042
Number of daily servings of vegetables	227 (69.6%)	112 (56.6%)	0.041	88 (60.3%)	47 (69.1%)	50 (78.1%)	42 (87.5%)	0.038
Number of daily servings of fruits	224 (68.7%)	134 (67.6%)	ns	92 (63.0%)	47 (69.1%)	54 (84.4%)	41 (85.4%)	0.034
Number of daily servings of fats	79 (24.2%)	94 (47.4%)	0.001	23 (15.7%)	23 (33.8%)	21 (32.8%)	12 (25%)	0.035
Daily fluid intake	191 (58.5%)	97 (48.9%)	0.036	84 (57.5%)	44 (64.7%)	39 (60.9%)	24 (50%)	0.041
Infant formulas and breastmilk can be replaced with cow’s milk, goat’s milk, or sheep’s milk past the age of 1 year	106 (32.5%)	73 (36.8%)	ns	29 (19.8%)	25 (36.7%)	23 (35.9%)	19 (39.6%)	0.025
Infant formulas and breastmilk can be replaced with plant-based beverages (such as rice or almond milk) past the age of 1 year	154 (47.2%)	107 (54%)	0.042	84 (57.5%)	36 (52.9%)	25 (39.1%)	9 (18.8%)	0.031
Fennel tea, fennel oil, and fennel-based pharmaceutical preparations	144 (44.1%)	109 (55.05%)	0.037	67 (45.9%)	36 (52.9%)	38 (59.4%)	11 (22.9%)	0.029
Recommended daily intake of juice for children aged 12–36 months	196 (60.1%)	124 (62.6%)	ns	115 (78.7%)	34 (50%)	27 (42.2%)	20 (41.7%)	0.032
Protein sources in a vegan diet	224 (68.7%)	41 (20.7%)	0.001	120 (82.8%)	47 (69.1%)	29 (45.3%)	28 (58.3%)	0.028
Sources of omega-3 fatty acids in a vegan diet	246 (75.4%)	79 (39.9%)	0.001	141 (95.6%)	44 (64.7%)	36 (56.3%)	25 (52.1%)	0.021
Recommended daily intake of dietary fiber for children aged 12–36 months	217 (66.5%)	106 (53.5%)	0.025	99 (67.8%)	48 (70.5%)	44 (68.8%)	26 (54.2%)	0.031
Sources of probiotic bacteria in a vegetarian diet	241 (73.9%)	97 (48.9%)	0.027	131 (89.7%)	48 (70.5%)	34 (53.1%)	28 (58.3%)	0.038
Do children raised on a vegetarian diet require iron supplements?	176 (53.9%)	128 (64.6%)	0.036	93 (63.7%)	32 (47.1%)	27 (42.2%)	24 (50%)	0.041
Do children raised on a vegetarian diet require calcium supplements?	201 (61.6%)	134 (67.6%)	0.041	84 (57.5%)	42 (61.7%)	32 (50.0%)	43 (89.6%)	0.039
Do children raised on a vegetarian diet require vitamin B12 supplements?	247 (75.7%)	121 (61.1%)	0.031	124 (84.9%)	52 (76.5%)	42 (65.6%)	29 (60.4%)	0.038
Types of nutritional deficiencies in children raised on a vegetarian diet	193 (59.25)	89 (44.9%)	0.031	93 (63.7%)	34 (50%)	32 (50.0%)	34 (70.8%)	0.026
Types of nutritional deficiencies in children raised on a vegan diet	178 (54.6%)	72 (36.4%)	0.028	67 (45.9%)	29 (42.6%)	42 (65.6%)	40 (83.3%)	0.037

ns—not significant.

**Table 4:** Odds ratios (95% confidence interval) in an analysis of differences in the consumption frequency of various foods recommended and not recommended in the model food ration between the experimental group and the control group, between different vegetarian groups, and between mothers with low and high nutritional knowledge scores.

	Experimental Group vs. Control Group			Lacto-Ovo-Vegetarian Group vs. Vegan Group			Low Nutritional Knowledge Score vs. High Nutritional Knowledge Score		
	OR	95% CI	*p*-Value	OR	95% CI	*p*-Value	OR	95% CI	*p*-Value
Juice	1.34 *	1.06–1.48	<0.05	1.46 **	1.19–1.67	<0.01	1.26 *	1.03–1.45	<0.05
Sweetened beverages	0.96	0.78–1.12	ns	1.31 *	1.06–1.54	<0.05	1.45 **	1.09–1.62	<0.01
Sweet snacks	0.94	(0.81–1.08)	ns	1.29 *	1.11–1.43	<0.05	0.94	0.82–1.07	ns
Sweets	1.06	0.89–1.23	ns	1.09	0.88–1.24	ns	1.13	0.93–1.27	ns
Natural dairy products or plain yogurt	0.78 *	0.63–0.97	<0.05	1.39 *	1.01–1.54	<0.05	0.71 *	0.61–0.88	<0.05
Breakfast cereals	1.11	0.93–1.30	ns	1.27 *	1.12–1.46	<0.05	1.29 *	1.07–1.41	<0.05
Fruits	1.34 *	1.11–1.48	<0.05	1.31 *	1.06–1.49	<0.05	1.04	0.89–1.27	ns
Vegetables	1.46 **	1.25–1.72	<0.01	0.93	0.81–1.09	ns	0.74 *	0.63–0.90	<0.05
Legumes	1.59 **	1.17–2.06	<0.01	0.81 *	0.71–0.94	<0.05	1.05	0.92–1.18	ns
Water	1.17	0.94–1.36	ns	1.03	0.94–0.15	ns	0.75	0.59–0.91	<0.05

* *p* < 0.05, ** *p* < 0.01 significance of differences in mean food consumption frequency (times/day) in Wilcoxon’s test (for two dependent samples); ns—not significant.

**Table 5:** Odds ratios (95% confidence interval) in an analysis of differences in the consumption frequency of various foods recommended in the model food ration between the experimental group and the control group, between different vegetarian groups, and between mothers with low and high nutritional knowledge scores.

	Daily Consumption of Cereals Other than Those Recommended in the Model Food Ration (Ref. [5] Servings)	Daily Consumption of Protein Foods Other than Those Recommended in the Model Food Ration (Refs. [4,5] Servings)	Daily Consumption of Vegetables Other than Those Recommended in the Model Food Ration (Ref. [5] Servings)	Daily Consumption of Fruits Other than Those Recommended in the Model Food Ration (Ref. [4] Servings)	Daily Consumption of Added Fats Other than Those Recommended in the Model Food Ration (Ref. [2] Servings)
Maternal nutritional knowledge score (n)					
Low (0–13)	0.97 (0.01–1.03)	0.92 (0.87–1.02)	1.07 (0.93–1.24)	1.09 (0.94–1.18)	0.72 ** (0.62–0.94)
Average (14–20)	0.78 * (0.91–0.93)	0.9 (0.71–1.03)	1.04 (0.92–1.11)	1.36 * (1.07–1.52)	0.94 (0.81–1.09)
High (21–26)	0.83 * (0.77–0.95)	0.92 (0.78–1.09)	1.27* (1.03–1.34)	1.17 (0.97–1.28)	0.91 (0.79–1.04)
**Type of diet**					
Vegetarian	0.84 * (0.72–0.98)	0.78 * (0.72–0.86)	1.11 (0.94–1.23)	1.21 (1.02–1.38)	0.84 * (0.67–0.97)
Control	1.46 ** (1.12–1.59)	1.20 (0.94–1.31)	0.77* (0.62–0.89)	0.93 (0.87–1.02)	1.09 (0.84–1.26)
**Vegetarian diet**					
Lacto-ovo-	0.97 (0.88–1.05)	1.12 (1.01–1.19)	1.08 (0.93–1.17)	1.29 * (1.11–1.32)	1.17 (1.05–1.26)
Lacto-	0.94 (0.83–1.03)	0.94 (0.87–1.09)	1.03 (0.89–1.19)	1.06 (0.92–1.12)	1.03 (0.92–1.25)
Ovo-	0.86 * (0.75–0.91)	0.81 * (0.72–0.96)	0.96 (0.84–1.09)	1.11 (1.02–1.2)	1.06 (0.92–1.27)
Vegan	0.75 * (0.63–0.89)	0.61 ** (0.49–0.86)	1.26 * (1.12–1.44)	1.34 * (1.12–1.51)	0.67 ** (0.48–0.79)

* *p* < 0.05, ** *p* < 0.01 level of significance assessed by Wald’s test; Deviations from the model food ration were most frequently noted in the vegan group, where the consumption of protein foods was insufficient (*p* = 0.021) and where 56.3% of the children consumed less than 2 servings of fat per day (*p* = 0.003). Vegetable consumption exceeded the recommended level (5 servings per day) only in the vegan group (OR 1.26; 95% CI 1.12–1.44, *p* < 0.05), whereas fruit consumption exceeded the recommended level in the lacto-ovo-vegetarian group (OR 1.29; 95% CI 1.11–1.32, *p* < 0.05) and the vegan group (OR 1.34; 95% CI 1.12–1.51, *p* < 0.01). Low maternal nutritional knowledge was linked with less frequent consumption of fats (*p* = 0.0.003), whereas high maternal nutritional knowledge was associated with significantly higher consumption of vegetables (OR 1.27; 95% CI 1.03–1.34, *p* < 0.05) and lower than recommended consumption of cereal products (OR 0.83; 95% CI 0.77–0.95, *p* < 0.05).

## Data Availability

Due to ethical restrictions and participant confidentiality, data cannot be made publicly available.
